# Comparative study to evaluate the voluntary acceptance of two liquid oral formulations of ciclosporin in dogs

**DOI:** 10.1186/s13620-018-0138-9

**Published:** 2018-12-29

**Authors:** Srinivas J. Kammanadiminti, Lori A. Carter, Wolfgang Seewald, Kelly P. Doucette

**Affiliations:** 1 Elanco Animal Health, 2500 Innovation Way, Greenfield, IN 26140 USA; 2 Stillmeadow Inc., 12852 Park One Drive, Sugar Land, TX 77478 USA; 3 Elanco Animal Health, Mattenstrasse 24a, 4058 Basel, Switzerland

**Keywords:** Atopic dermatitis, Dog, Ciclosporin, Acceptance, Consumption, Prehension, Palatability

## Abstract

**Background:**

The purpose of this study was to determine and compare the voluntary acceptance of two oral liquid formulations of ciclosporin, investigational Atopica® oral solution (Elanco Animal Health) and Cyclavance® Oral Solution (Virbac), when given orally via syringe or offered freely after mixing with food to dogs.

Twenty-five adult mixed breed dogs were selected for this two-phase study. In Phase 1, 12 (Group I) and 13 (Group II) dogs received Atopica® oral solution and Cyclavance® Oral Solution, respectively, daily for 7 days via an oral syringe. After a 3-day washout period, the dosing was switched for a further 7 days. For Phase 2, dosing was by acceptance from freely offered test article mixed in a small amount of food, approximately 6 h after the routine morning feeding. During the first part of this phase, normal daily ration of food offered in the morning was continuously left in the cage. Group I was offered Atopica® oral solution and Group II was offered Cyclavance® Oral Solution mixed with ~ 25 g of food for 3 days. After another 2-day washout period, the test articles were switched for another 3 days but the animals received food for only 1 h in the morning. Five hours after the food was removed, the test articles with food were offered in the same manner as in the first part of Phase 2. Animals were also monitored for adverse events (AEs).

**Results:**

During Phase I, voluntary acceptance rates of 100 and 98.9% were noted for Atopica® oral solution and Cyclavance® Oral Solution, respectively. The corresponding immediate prehension rates during Phase 2 (Period 1) were 61.1 and 56.4%, respectively. During Phase 2 (Period 2), the immediate prehension rates were 69.2, 69.4 and 92.0% for Atopica® oral solution, Cyclavance® Oral Solution and the positive control (DYNE®; High Calorie Liquid Dietary Supplement), respectively. Two adverse events of diarrhea and vomiting, with a probable relationship to the test articles, were reported.

**Conclusion:**

There was no significant difference in acceptance of the two oral ciclosporin solutions, the investigational Atopica® oral solution (Elanco) and Cyclavance® (Virbac) for dogs.

**Electronic supplementary material:**

The online version of this article (10.1186/s13620-018-0138-9) contains supplementary material, which is available to authorized users.

## Introduction

Canine atopic dermatitis (AD) is believed to be a genetically predisposed chronic inflammatory and pruritic dermatopathy manifested due to hypersensitivity to environmental allergens [1]. The incidence of AD in the general canine population is unknown; however, it is thought that the condition is on the rise due to either advancement in diagnosis or increase in prevalence of the disease. In an early study conducted in Southern England, canine AD was diagnosed in 78% of 280 dogs, of which 85–90% were sensitive to house dust mites and/or forage (storage) mites [2]. As per a recent report by Hsiao et al., nearly 10–15% of the canine population is affected by AD [3]. Secondary conditions include scaling, hyperpigmentation, pyoderma, bacterial overgrowth, allergic otitis externa, etc., further deteriorating the poor skin condition. Therefore, it is imperative to achieve control of pruritus, inflammation and infection in canine AD [4].

Ciclosporin has been in use in humans since 1983 for the management of AD because of its powerful immunosuppressant activity to prevent rejection of transplanted organs [5]. In 2003, the FDA approved the use of oral ciclosporin capsules (Atopica®; Elanco Animal Health) in veterinary medicine for the treatment of canine atopic dermatitis [6]. Subsequently, Atopica® oral solution for cats was also approved for feline indication. In Europe, several oral liquid ciclosporine products such as Cyclavance, Modulis and Sporimune are available; however, apart from for Cyclavance, acceptability of other products by dogs is unknown. The International Committee on Allergic Diseases of Animals (ICADA) guidelines for the treatment of canine AD recommends oral ciclosporin for the reduction of pruritus and skin lesions. The treatment is recommended until clinical signs are controlled, which may take 4–6 weeks [7].

The ciclosporin formulations in all Atopica® products for veterinary use are identical to Sandimmun Neoral® 100 mg/ml Oral Solution marketed by Novartis Pharma for human use. It is an ultramicronized liquid preparation of ciclosporin that forms a microemulsion upon contact with aqueous fluids which was found to result in lower inter- and intra-subject variability in exposure [6].

Ease of administration is an important consideration during formulation development of a drug. Therefore, formulations such as solutions, suspensions, emulsions, capsules and granules have been developed for ease of treatment administration [8]. The capsule formulation of ciclosporin for dogs is widely accepted by prescribers as a treatment for AD [9], allowing ease of administration. However, there can be instances where concerns related to prehension with a capsule formulation may arise in the long term [10]. Due to the chronic and recurring characteristics of AD, and the fact that it severely decreases the quality of life of animals [11], there is a need to have a liquid formulation with the goal of achieving a high compliance and acceptance of the prescribed drug. Acceptance is another important factor that affects compliance, especially for chronic therapy [8]. Recent studies comparing the Atopica® capsule formulation of oral ciclosporin with a generic liquid oral formulation showed that both formulations were bioequivalent; nevertheless, study animals preferred the liquid over the capsule formulation [10, 12]. Consequently, it was decided to develop a liquid formulation for dogs to provide an alternative to the currently marketed Atopica® Capsules.

The objective of this study was to determine the acceptance of investigational Atopica® oral solution for dogs by comparing it with that of Cyclavance® Oral Solution when given orally via syringe to dogs (Phase 1) and when offered freely after mixing with a small amount of food (Phase 2).

## Methods

This single-site, randomized, controlled, two-phase, partial cross-over study was performed at Stillmeadow Inc., Sugar Land, TX, USA and was compliant with the Animal Welfare Act Regulations and the standard procedures at the test facility. Study was approved by the Test Facility’s IACUC under Animal Use Protocol number 19426–15. Acceptance of the test articles was evaluated at the recommended dosage of 5 mg/kg body weight.

### Study design

#### Animals

Thirty adult, mixed-breed healthy dogs (as determined by physical examination, fecal analysis and acceptable clinical pathological evaluation) were acclimated to the dosing procedure of administration via syringe for 14 days using DYNE®; High Calorie Liquid Dietary Supplement for Dogs.

From these animals, 25 (mixed sex distribution) dogs that were satisfactorily acclimated to the dosing procedure were selected for inclusion in the study and were randomized based on body weight into two groups: Group I (*n* = 12) and Group II (*n* = 13). All dogs were aged > 6 months and weighed between 7.7 and 24.1 kg. Animals were housed, managed and fed as per facility procedures. Water was given ad libitum.

Animals were weighed twice during acclimation. General health observations were conducted twice daily throughout the study and included, but were not limited to, observations of general physical appearance and behavior. Approximately 5 mL of blood was collected for clinical chemistry and complete blood count (CBC) from each animal by jugular or cephalic venipuncture after overnight fasting during the acclimatization phase.

#### Test articles

The articles tested were Sandimmun Neoral® 100 mg/ml oral solution (Novartis, Delpharm Huningue, France) (referred to as Atopica® oral solution) and Cyclavance® 100 mg/ml oral solution (Virbac Espana, Esplugues de Llobregat, Spain). Both test articles were administered orally (via syringe or mixed with a small amount of food). DYNE®; High Calorie Liquid Dietary Supplement (Lambert Kay) mixed with a small amount of food was administered as the positive control.

**Fig. 1:**
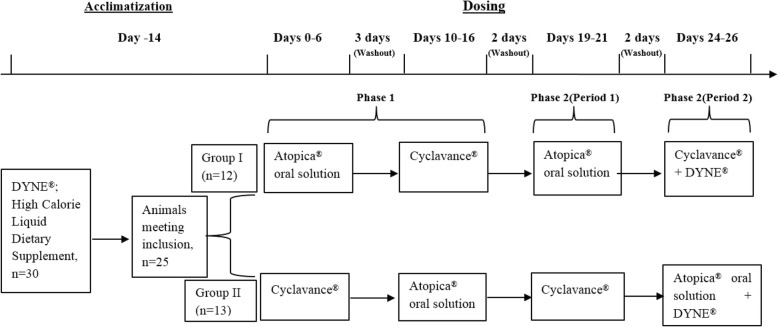
Experimental design of the study. During acclimation period, dogs received nutritional supplement via syringe for training purpose. All animals were administered 5 mg/kg (dose volume 0.05 mL/kg) of the test articles via syringe directly in the dog’s mouth, at least 2 h before feeding in the morning. In Phase I & II, Group I and Group II animals received one of the test articles as shown

Dosing was conducted in two phases. Figure 1 provides a basic outline of the experimental design.

#### Phase 1

All animals were administered 5 mg/kg (dose volume 0.05 mL/kg) of the test articles via syringe, at least 2 h before feeding in the morning. Test articles were administered directly in the dog’s mouth. Group I received Atopica® oral solution and Group II received Cyclavance® Oral Solution daily for 7 days. After a 3-day washout period, dosing was switched for the groups and treatment administration was continued for a further 7 days. A 2-day washout period followed completion of Phase 1 of the study.

#### Phase 2

For this phase, dosing was by acceptance from freely offered test article mixed in a small amount of food, approximately 6 h after the routine morning feeding. All animals were offered the appropriate test article (5 mg/kg; 0.05 mL/kg) mixed with approximately 25 g of food. In the first part, when the daily ration offered in the morning was not removed anytime, Group I was offered Atopica® oral solution and Group II was offered Cyclavance® Oral Solution mixed with food for 3 days. Following a 2-day washout period, the test articles were switched for a further 3 days. The feeding schedule for this second part of Phase 2 was that the animals received routine daily food portion for only 1 h in the morning. Five hours after the food was removed, the test article with a small amount (~ 25 g) of food was offered in the same manner as in the first half of Phase 2. Because of this, Phase 2 was no longer a true cross-over design and data from both parts of the phase were not combined for analysis.

#### Evaluation of acceptance

For Phase 1, acceptance was evaluated by the ease of test article administration. Intake was classified as Score 1 - voluntary acceptance (syringe was easily inserted into the dog’s mouth combined with dog’s willingness to swallow the test article) or Score 2 - forced administration (need for strong animal handling to insert the syringe into the dog’s mouth and administer the article at the back of the throat/into the cheek or need for restraint to ensure swallowing). In both phases, a single technician dosed and evaluated all dogs on each day. Multiple technicians were involved in the study and the same individual did not do evaluation on every day.

For Phase 2, acceptance was evaluated by prehension (animal voluntarily took the food with test article into the mouth within 2 s or 1 min) and consumption of the food with test articles. Prehension was classified as Score 1 - immediate (within 2 s) or Score 2 - delayed (eaten after 2 s but within 1 min). If prehension occurred, dogs were observed for an additional 5 min to register whether the article was swallowed or spat out. A score of 3 meant no prehension or consumption by 1 min, but with complete dosing (all food consumed) within approximately 5 min. Food (with the test articles) consumption was classified as total, partial or none. For the purpose of statistical evaluation, no prehension or consumption by 1 min with incomplete dosing (no food consumed even by the additional 5 min) was classified as a score of 4.

#### Positive control assessment

During Period 2 of Phase 2 of the study, all animals were offered the positive control, DYNE®; High Calorie Liquid Dietary Supplement for Dogs mixed with a small amount of food. The offering was made each day after the test article acceptance assessment. An assessment of the positive control acceptance was conducted with the same scoring system as test article acceptance assessment for Phase 2.

#### Tolerance assessment

In addition to the general health observations conducted twice daily, clinical observations were conducted prior to dosing and 30 min post dosing. Clinical observations included but were not limited to observations of general physical appearance and behavior, abnormalities of food and water consumption and appearance of urine and feces. Any abnormal findings were recorded, including the severity, duration, frequency, etc.

Complete physical examinations by the veterinarian were conducted during acclimatization and 3 days after offering the final dose (Additional file 1: Table S5).

Furthermore, all animals were weighed before dosing and at the end of the study. Approximately 5 mL of blood was collected before the dosing and the day after offering the final dose for hematology and biochemistry analyses. All parameters were analyzed at Antech Diagnostics, Dallas, TX, USA.

#### Statistical analysis

Frequency counts were calculated for each treatment in phase 1 (score 1 & 2) and in each period of phase 2 (scores 1–4). Animal average rates were compared between treatments in phase 1 using the Wilcoxon paired-sample test and in each period of phase 2 using the Mann-Whitney U test. Daily acceptance rates were analyzed with a generalized linear mixed model, with the following fixed effects: treatment; period; day within period; and subject as a random effect. All calculations were carried out using the software SAS®, Version 9.2.2 (SAS Institute Inc., Cary, NC).

## Results

**Fig. 2:**
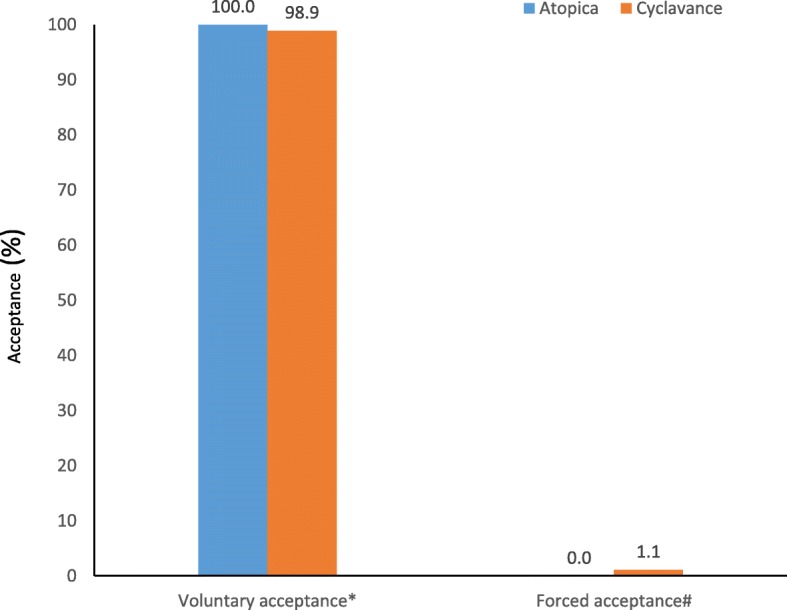
Acceptance of Atopica® and Cyclavance® solutions administered via syringe. *Voluntary acceptance: Syringe is easily inserted into mouth combined with willingness to swallow; #Forced acceptance: Need for strong animal handling to insert the syringe into the dog’s mouth and administer the article at the back of the throat/into the cheek or need for restraint to ensure swallowing

During Phase 1, when the administration was via syringe, a total of 350 data points were collected (25 animals receiving the 2 test articles for 7 days). In this Phase high voluntary acceptance rates of 100 and 98.9% were observed for Atopica® oral solution and Cyclavance® Oral Solution, respectively with no significant difference (*p* = 0.5; Fig. 2).

**Fig. 3:**
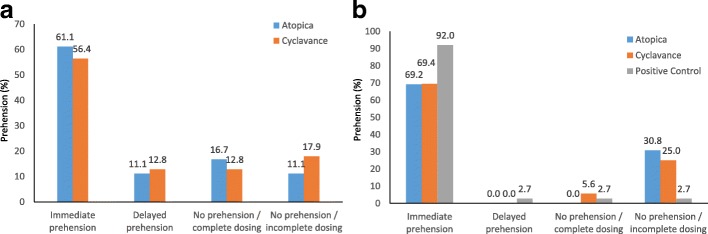
Overall acceptance of Atopica® and Cyclavance® oral solutions when mixed with a small amount of food. **a** Daily ration of food was available throughout the 6 h preceding the test article administration. **b** Daily ration of food removed after an hour and acceptance tested 5 h later. Acceptance of Atopica and Cyclavance after mixing in food. Immediate prehension: Food and test article taken into the mouth within 2 s; Delayed prehension: Food and test article taken into the mouth after 2 s; No prehension: Test article and food mixture remaining after 1 min; Complete dosing- food was taken within an additional 5 min; Incomplete dosing - no food was consumed even by the additional 5 min. DYNE®; High Calorie Liquid Dietary Supplement was the positive control in the scenario of food removed and acceptance tested 5 h later

Acceptance rates by prehension and consumption for both the test articles in Phase 2, Period 1 are shown in Fig. 3a. No significant difference in either the prehension rates or consumption were observed for Atopica® oral solution and Cyclavance® Oral Solution (*p* = 1.0000). Immediate prehension rates of 61.1% & 56.4% and delayed prehension rates of 11.1% & 12.8% were observed for Atopica® and Cyclavance®, respectively. Even when there was no prehension (test articles in food not eaten within 1 min), consumption rates (eaten were also similar between the test articles with a complete dosing (full consumption) of 16.7% & 12.8% and incomplete dosing (partial consumption) of 11.1% & 19.7%, respectively, for Atopica® and Cyclavance®.

Figure 3b demonstrates the acceptance rates by prehension and consumption for both the test articles in study Phase 2, Period 2. The immediate prehension rate for Atopica® was comparable to that of Cyclavance® (*p* = 0.9765). Immediate prehension rates of 69.2, 69.4% & 92% and delayed prehension rates of 0, 0% & 2.7% were observed for Atopica®, Cyclavance® and positive control, respectively. Consumption rates (food taken within additional 5 min) were also similar between the test articles with a complete dosing (full consumption) of 0, 5.6% & 2.7% and incomplete dosing (partial consumption) of 30.8, 25% & 2.7%, respectively, for Atopica®, Cyclavance® and positive control.

**Fig. 4:**
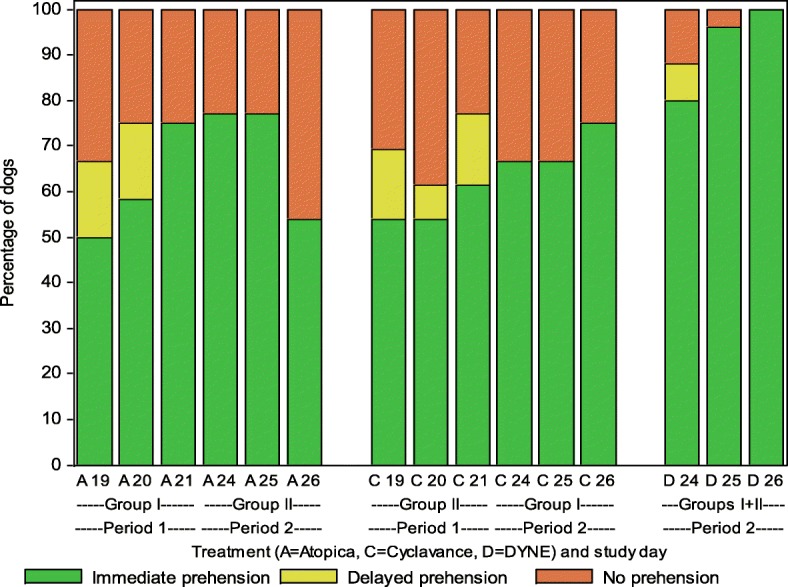
Daily acceptance rates of Atopica® and Cyclavance® offered with small amount of food. Period 1: Food offered in the morning left in the cage continuously; Period 2: Food offered in the morning only for 1 h and acceptance testing was done approximately 5 h after removal of food. Testing for each period was for 3 days with a 2-day washout in between

Daily acceptance rates of the two products offered with small amount of food are presented in Fig. 4. Statistical analysis revealed no significant differences in the immediate or delayed prehension rates among a) test articles, b) any day within each period or c) between the two periods of Phase 2. However, a significant effect of subject was noted (Table 1).

**Table 1:** Significant effects on acceptance rates observed between Atopica® and Cyclavance® offered in small amount of food

Response	Effect	*p*-value
Immediate prehension	treatment	0.6813
	period	0.0738
	day	0.4762
	subject	<.0001***
Immediate or delayed prehension	treatment	0.7947
	period	0.7932
	day	0.7561
	subject	<.0001***

*** denotes statistical significance

Voluntary acceptance was tested when Atopica® and Cyclavance® solutions were mixed with ~ 25 g of food. In Period 1 the daily ration offered in the morning was left in the cage and testing was done 6 h later. Subsequently, in Period 2, food was offered only for an hour and acceptance tested 5 h after food removal. N = 12–13/group. Testing for each period was for 3 days with a 2-day washout in between.

Both formulations were well tolerated in the study animals with no serious adverse event (SAE) reported. Four adverse events (AEs) were reported during Phase 2 of the study. Of these, two AEs of vomiting (moderate) and diarrhea (mild) had a probable relationship to the test articles, whereas the remaining two AEs of otitis externa on the left ear and corneal crystals in both eyes were unlikely to be related to the test articles. Mild AE of slight diarrhea was noted on day 25 pre-dose observation in one Group II animal. Moderate AE of vomiting was observed in another animal on day 26 following administration of Sandimmun® Neoral (Atopica). Both these animals also received the positive control, DYNE® Dietary Supplement in this same period and Cyclavance previously on days 19–21.

No significant effects on body weight, blood chemistry or clinical chemistry parameters were noted in any animal .

### Additional information

Supplementary information (File name: Atopical Oral Solution for dogs_Supplementary Information.pdf) has individual animal acceptance data at different phases of the study (Additional file 1: Tables S1-S3), body weights (Additional file 1: Table S4), physical examination record (Additional file 1: Table S5), hematology (Additional file 1: Table S6) and serum chemistry (Additional file 1: Table S7) parameters.

## Discussion

This study compares the acceptance of two liquid formulations of ciclosporin, an investigational Atopica® oral solution with that of an approved generic product, Cyclavance® Oral Solution.

Ciclosporin is currently one of the most effective treatments for canine AD [9]. The ciclosporin capsule formulation (Atopica®, Elanco Animal Health) was first approved for the management of canine AD. Amongst the commercially available dosage forms, oral liquids are the simplest, convenient and economical dosage forms for treatment of chronic diseases [13]. Therefore, a liquid oral formulation of ciclosporin is being registered for the treatment of canine AD. Recently Navarro et al. described superior acceptance of a generic ciclosporin (Cyclavance® by Virbac, France) over Atopica® capsules for dogs (Elanco Animal Health, formerly, Novartis Animal Health) [10]. The investigational formulation used in the current study was Sandimmun® Neoral for human use that was identical with the formulations in the approved capsules for dogs and in Atopica® Oral Solution for cats. Based on the success of these approved Atopica® products, Elanco Animal Health is now developing the same formulation for dogs as oral liquid formulation as a proven alternative to oral capsule formulation. Accordingly, this study compared the acceptance of two liquid formulations of ciclosporin, Sandimmun Neoral® Solution (proposed investigational Atopica® oral solution for dogs, Elanco Animal Health) and Cyclavance® Oral Solution (Virbac, France).

The acceptance tests were run over a period of 26 days and included two phases. In Phase 1, acceptance was measured by the ease of administration of test articles via syringe and in Phase 2 by prehension and consumption of test articles mixed in a small amount of food. In this study, animals were randomized based on body weight to minimize bias. Variability in mean body weight of the groups was within 20%. Phase 1 was a classical crossover design with test articles switched for the two groups with a 3-day washout period, whereas Phase 2 was not a crossover design but consisted of two separate, parallel designs.

Phase 1 of this study may not be considered a true palatability evaluation since the products in syringe were inserted into the animal’s mouth, as opposed to offering freely. However this phase complied with many other recommendations of the current guidelines on conduct of palatability study [14]. For example, the guideline states “For daily treatments lasting more than 14 days, seven daily consecutive administrations should generally be sufficient”. Accordingly, this was conducted with 7-day dosing with both test articles. Also the sample size of 25 animals is sufficient to draw valid conclusions if the product is administered at least twice. In this phase, with 350 tests conducted over 2-week period, the voluntary acceptance rate of Atopica® oral solution was similar to that of Cyclavance®, 100 and 98.9%, respectively. With 140 tests, Navarro et al. reported acceptance of 99% for Cyclavance® and only 31% for Atopica® capsules [10]. Our study corroborated the high acceptance of Cyclavance® while clearly suggesting that Atopica® oral solution is also equally well accepted. Unlike flavored tablets, capsules typically have to be inserted at the back of the animal’s mouth and a low voluntary acceptance from hand in that study was not surprising. It is also important to note that the previous study evaluated this acceptance of Cyclavance® via syringe only in Beagle dogs while our study involved mixed breeds that are more relevant population in the clinical scenario. Nonetheless data from both studies suggest that Atopica® solution can have better acceptance over capsules and demonstrated there was no significant difference between the two liquid formulations when administered via this standard clinical method of administration. Washout period of 2–3 days in between the change of treatments was based on the previous publication [10] and also author’s empirical experience which shows that this period would help avoid the ‘memory effect’ of the previous product.

Atopica® is not to be administered with a standard meal, however, similar to that reported in previous publication [10], Phase 2 was conducted by mixing the test articles in ~ 25 g of food (representing < 10% of standard daily meal) and offering freely in the cage, about 6 h after the daily ration was fed in the morning for three consecutive days. Initially, Phase 2 was also planned to be a cross-over design with test articles switched for the two groups and generate 350 data points as in Phase 1. However, in the first part (Period 1), acceptance rates for both products were unexpectedly lower (immediate prehension rates of 61.1 and 56.4% for Atopica® and Cyclavance® solutions, respectively) than previously reported for Cyclavance® solution (90.6%) [10]. It was hypothesized that availability of food continuously during the testing period contributed to this observation. Supporting this contention was the observation that some dogs were slow eaters that did not eat much food immediately when offered in the morning (~ 8 am). Perhaps these dogs would have consumed food just before the testing time (~ 2 pm) and hence were not interested to accept the test articles offered with small amount of the same food. Hence to test this hypothesis, the study was amended by offering the daily ration only for an hour in the morning and acceptance was tested about 5 h after removal of food (Period 2). Surprisingly, only a slight increase in acceptance but no statistically significant difference was observed compared to Period 1 (Fig. 4 and Table 1) with immediate prehension rates of 61.1 and 69.2% for Atopica® oral solution and 56.4 and 69.4% for Cyclavance®, respectively for periods 1 and 2. However, 92% acceptance of the positive control validated the low acceptance of test articles in period 2. Lack of difference in acceptance was more conspicuous if immediate and delayed prehensions were combined, which resulted in 72.2% & 69.2% for Atopica® solution and 69.2% & 69.4% for Cyclavance® solution, under the ‘fed’ (period 1) and ‘semi-fasted’ (period 2) conditions, respectively (interestingly, there was no delayed prehension, suggesting ‘all’ or none’ phenomenon in this ‘semi-fasted’ condition). The precise feeding conditions were not apparent from the previous comparative study of Cyclavance® solution and Atopica® capsules [10] but it is likely that the food was continuously made available (standard husbandry practice at animal facilities), similar to that in the period 1 of our study. Hence the causes of lower acceptance in our study were not clear. A careful analysis of acceptance by individual animals revealed that six animals (3 each in Groups 1 and 2) consistently refused to accept any test article in food under either condition. Out of these 6, four animals (two in each group) failed to accept the positive control as well. If these animals are excluded, the difference between the two periods became significant (*p* = 0.0493). However, the limitation of lower samples (*N* = 12–13/group) to draw reliable conclusion is acknowledged. Previous observation [10] of significantly lower acceptance of capsules was not unexpected particularly when buried in palatable kibbles (14.4% immediate prehension and 2.2% total consumption). Treatment with liquid formulation can increase the acceptance rate when compared to a capsule formulation as the taste is masked by the palatability of the food in the former whereas the dog can eat around the capsule thereby decreasing acceptance for the latter. Even when taken into the mouth, some dogs apparently spat out the capsules, perhaps due to the non-anticipation of the presence of inert capsule in the middle of a palatable food.

Overall, it is clear that in this study, oral solutions of both Atopica® and Cyclavance® were accepted equally well in each of the three tested conditions.

Phase 1 followed the EMA guidelines on palatability testing in terms of sample size and the duration of testing. However, testing the acceptance of the product is not according to the formal palatability studies due to the fact that product was administered in a syringe by holding the animal and acceptance was assessed based on the ‘ease’ of administration which is prone to be subjective. To minimize this factor, the same person has evaluated all animals but the subjective nature cannot be ruled out completely. While this design followed the previous work [10], in a true palatability study, the product is offered in an empty bowl or trough, on the ground or by hand. Even the Phase 2 where test articles were administered in a very small quantity of food, can be a true palatability study because the guideline [14] states ‘The palatability of the tested product should be assessed without food to avoid any effect of palatability linked to the food composition’. Even though, test articles were mixed only with a very small portion of standard food (< 10% of daily ration), it is possible that food might have impacted the test article flavor and hence, no palatability claim can be made based on this data. The low acceptance of both the test articles in Period 1 of Phase 2 is surprising and lead to test the positive control in period 2. Reasons for the consistent low acceptance in both the periods is difficult to interpret and perhaps testing this with different types of food (brands of different flavor, canned food etc.) could provide some answers to this observation. Nonetheless, the objective of this study is to compare the relative acceptance of Atopica and Cyclavance and while no difference was observed between the test articles, both had < 80% acceptance required by the guideline [14] for palatablity claim when combined with food. However, Atopica label indicates that the product should be administered before or after food and hence the > 80% acceptance in Phase I is encouraging.

There was no significant change in the bodyweight of dogs in both groups throughout the study, demonstrating that either test article was well tolerated.

Earlier studies have established the safety of ciclosporin in dogs, with most of the AEs being manageable without any additional need of medical intervention [10]. In this study, four AEs were reported during Phase 2. Of these, two AEs of slight diarrhea (predose observation on day 25) and extreme vomiting (post dose observation on day 26) had a probable relationship to the test articles and were classified as mild and moderate in severity, respectively, based on the single instance in a single animal. As the above AEs occurred after multiple administrations of both test articles (due to cross-over design), it was not possible to attribute these effects to a specific test article. Further, these digestive disturbances are anticipated side effects with ciclosporin products. During the physical examination conducted post study completion, AEs of otitis externa on the left ear and corneal crystals in both eyes were noted in two animals. Both these conditions were unlikely to be related to the test articles.

Overall, there were no significant differences in the acceptance and tolerance between the two tested ciclosporin liquid formulations. These results indicate that the Atopica® oral solution (Elanco Animal Health) is accepted well by dogs and presents an alternative to currently marketed Atopica® capsules for dogs.

## Conclusion

In this study, the acceptance of investigational Atopica® oral solution (Elanco Animal Health) and Cyclavance® Oral Solution (Virbac) was comparable, with no significant differences in voluntary acceptance, prehension and consumption in dogs.

## Additional file

Additional file 1: Atopical Oral Solution for dogs_Supplementary Information. (DOCX 1754 kb)

## Abbreviations

AD: Atopic dermatitis
AE: Adverse event
CBC: Complete blood count
QOE: Quality of the evidence
SAE: Serious adverse event
SOP: Standard operating procedure
