# Supplementary material for: Comparative study to evaluate the voluntary acceptance of two liquid oral formulations of ciclosporin in dogs
Source: Ir Vet J. 2018 Dec 29;71:27. doi: 10.1186/s13620-018-0138-9 (PMC6310966; doi:10.1186/s13620-018-0138-9)
Supplement: Supplementary file 1 — Atopical Oral Solution for dogs_Supplementary Information. (DOCX 1754 kb) [file 13620_2018_138_MOESM1_ESM.docx]

**Table S1: Acceptance in Phase I- Individual Animal Data**


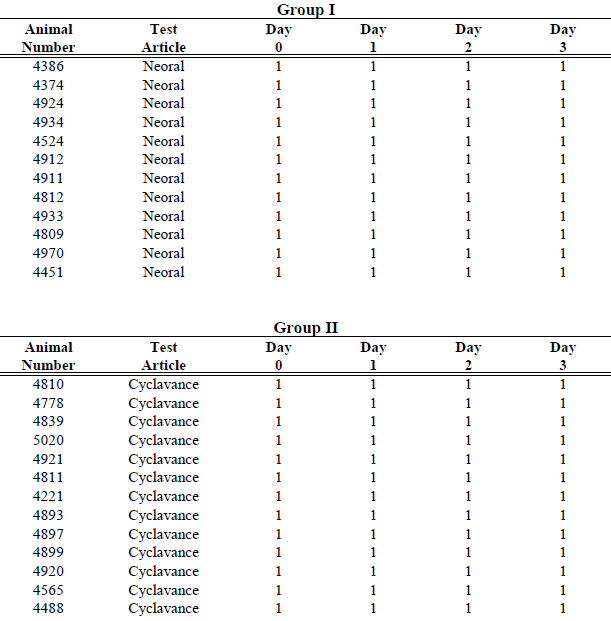


1. Voluntary acceptance- Syringe is easily inserted into mouth combined with willingness to swallow
2. Forced acceptance- Need for strong animal handling to insert the syringe into the dog’s mouth and administer the product at the back of the throat/into the cheek or need for restriant to ensure swallowing

**Table S1: Acceptance in Phase I- Individual Animal Data (continued)**


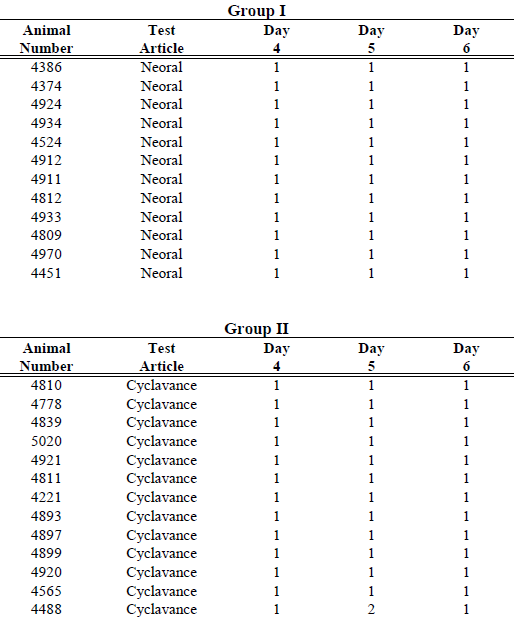


1. Voluntary acceptance- Syringe is easily inserted into mouth combined with willingness to swallow
2. Forced acceptance- Need for strong animal handling to insert the syringe into the dog’s mouth and administer the product at the back of the throat/into the cheek or need for restriant to ensure swallowing

**Table S1: Acceptance in Phase I- Individual Animal Data (continued)**


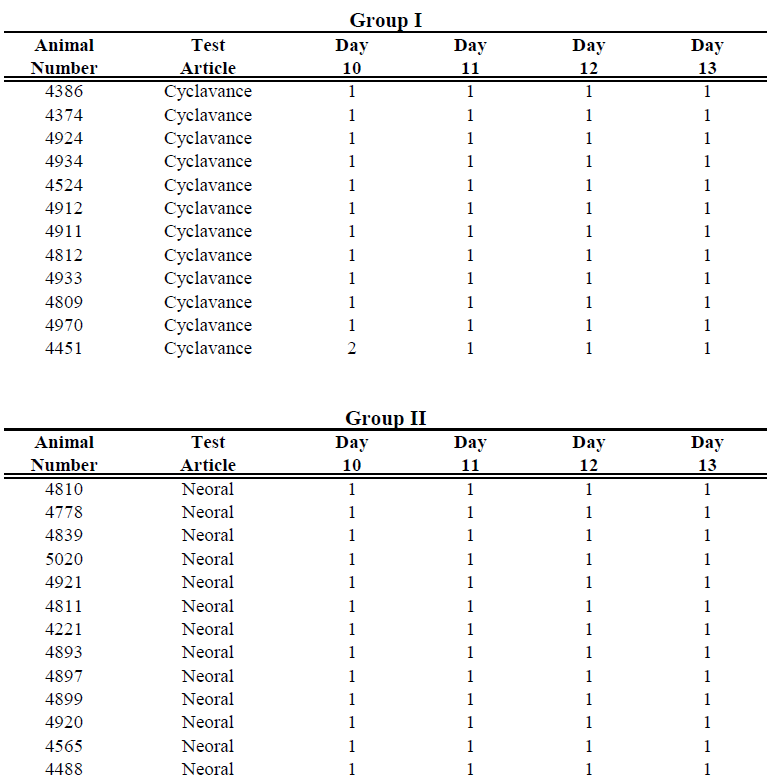


1. Voluntary acceptance- Syringe is easily inserted into mouth combined with willingness to swallow
2. Forced acceptance- Need for strong animal handling to insert the syringe into the dog’s mouth and administer the product at the back of the throat/into the cheek or need for restriant to ensure swallowing

**Table S1: Acceptance in Phase I- Individual Animal Data (continued)**


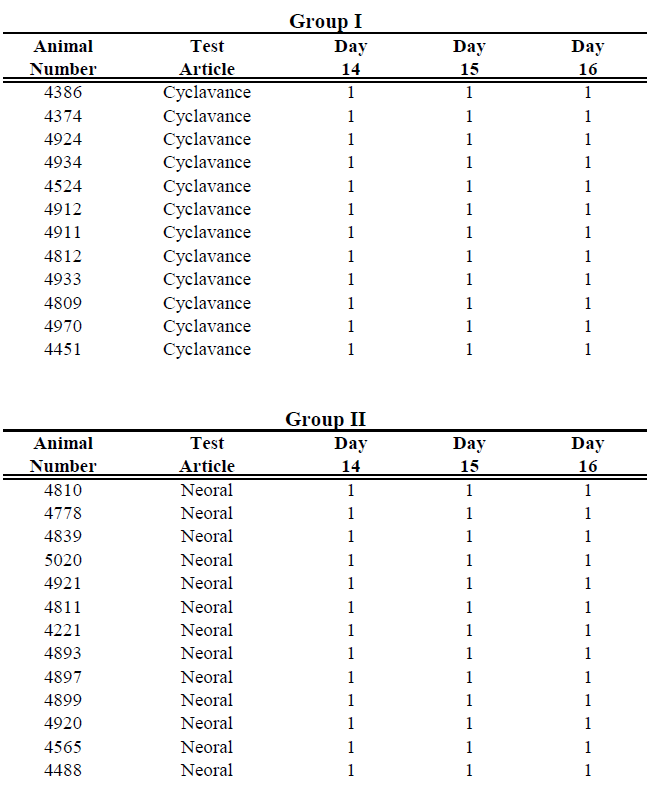


1. Voluntary acceptance- Syringe is easily inserted into mouth combined with willingness to swallow
2. Forced acceptance- Need for strong animal handling to insert the syringe into the dog’s mouth and administer the product at the back of the throat/into the cheek or need for restriant to ensure swallowing

**Table S2: Acceptance in Phase II (Period 1)- Individual Animal Data**


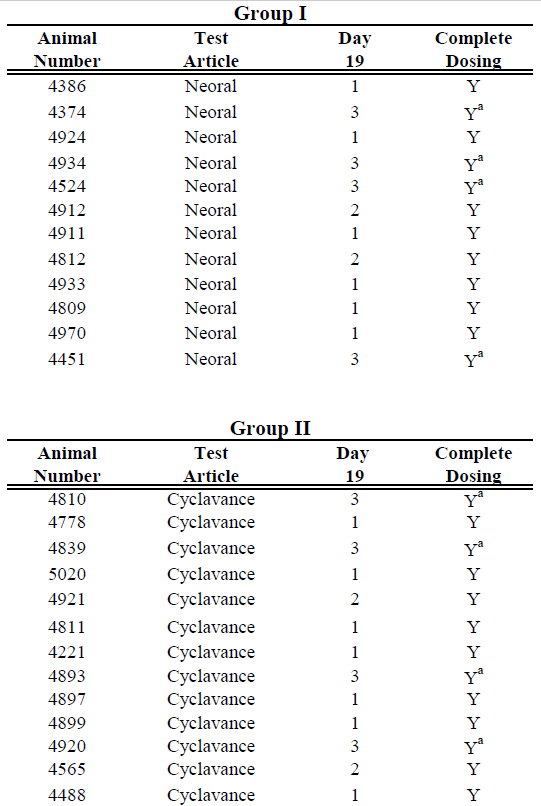


| 1 - Immediate Prehension - food and test article taken into the mouth within two seconds.  2 - Delayed Prehension - food and test article taken into the mouth after two seconds |
| --- |
| 3 - No Prehension - test article and food mixture remaining after one minute |
| Y - Yes |
| Ya - Yes; consumed after one minute |

**Table S2: Acceptance in Phase II (Perod 1)- Individual Animal Data (continued)**


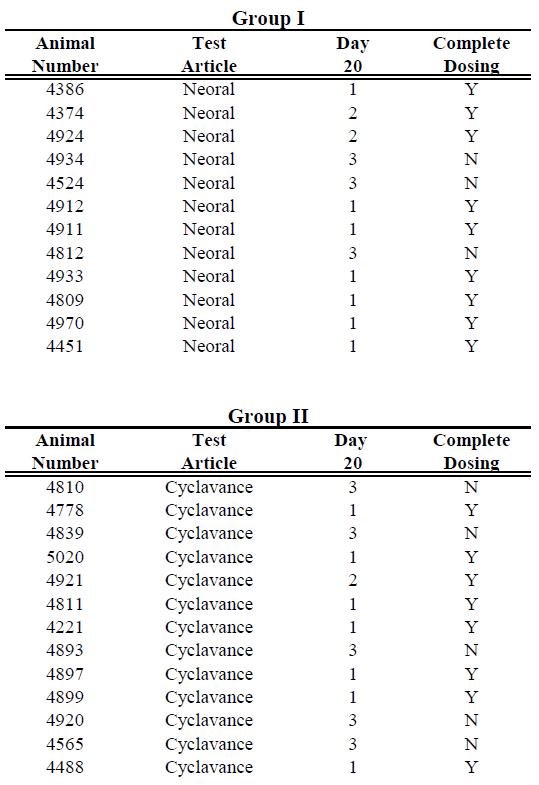


| 1 - Immediate Prehension - food and test article taken into the mouth within two seconds.  2 - Delayed Prehension - food and test article taken into the mouth after two seconds |
| --- |
| 3 - No Prehension - test article and food mixture remaining after one minute |
| Y - Yes |
| Ya - Yes; consumed after one minute |

**Table S2: Acceptance in Phase II (Perod 1)- Individual Animal Data (continued)**


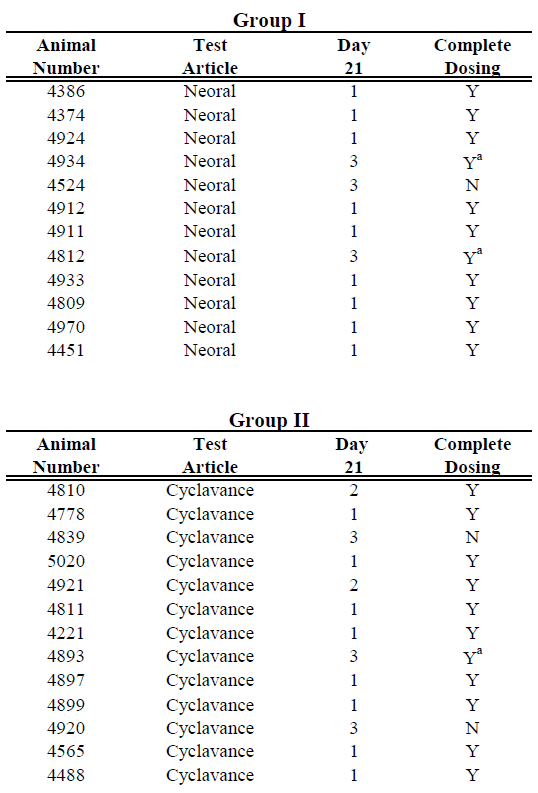


| 1 - Immediate Prehension - food and test article taken into the mouth within two seconds.  2 - Delayed Prehension - food and test article taken into the mouth after two seconds |
| --- |
| 3 - No Prehension - test article and food mixture remaining after one minute |
| Y - Yes |
| Ya - Yes; consumed after one minute |

**Table S3: Acceptance in Phase II (Perod 2)- Individual Animal Data**


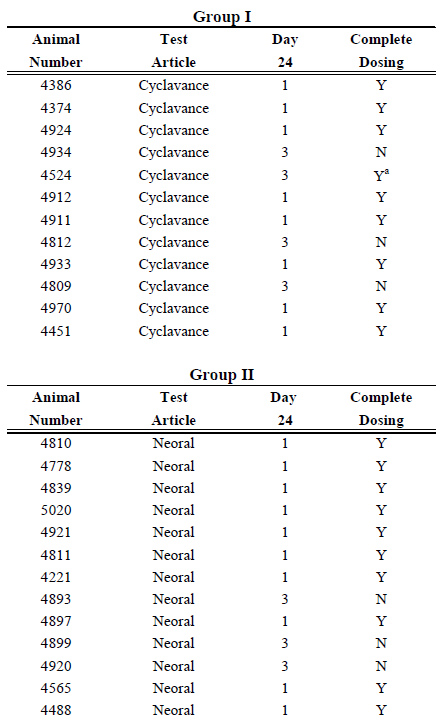


| 1 - Immediate Prehension - food and test article taken into the mouth within two seconds.  2 - Delayed Prehension - food and test article taken into the mouth after two seconds |
| --- |
| 3 - No Prehension - test article and food mixture remaining after one minute |
| Y - Yes |
| Ya - Yes; consumed after one minute |

**Table S3: Acceptance in Phase II (Perod 2)- Individual Animal Data (continued)**


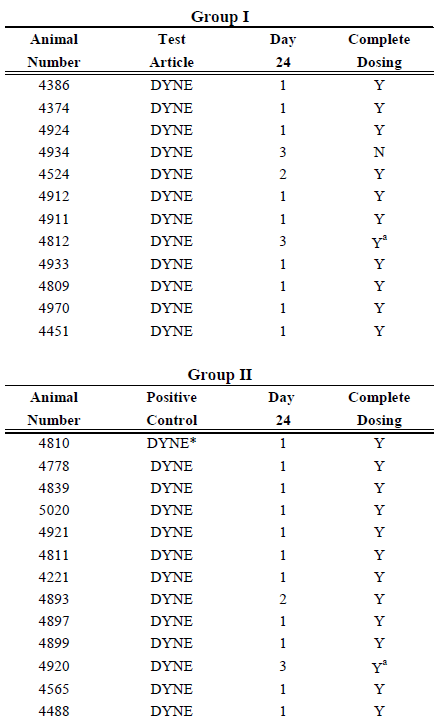


| * DYNE - Dyne High Calorie Liquid Diet Supplement |
| --- |
| 1 - Immediate Prehension - food and test article taken into the mouth within two seconds.  2 - Delayed Prehension - food and test article taken into the mouth after two seconds |
| 3 - No Prehension - test article and food mixture remaining after one minute |
| Y - Yes |
| Ya - Yes; consumed after one minute |

**Table S3: Acceptance in Phase II (Perod 2)- Individual Animal Data (continued)**


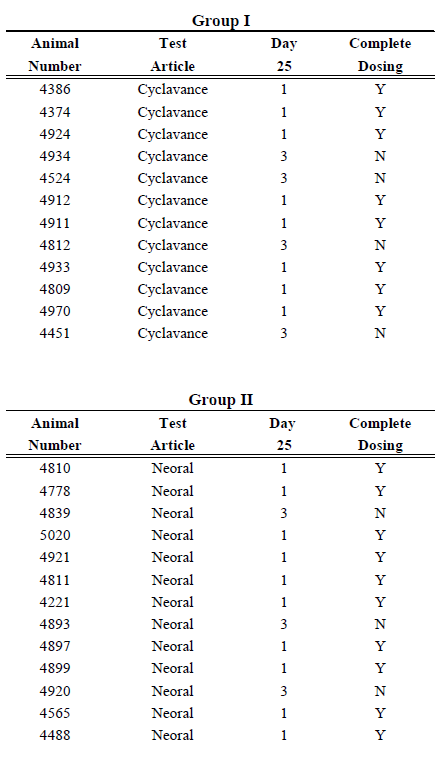


| 1 - Immediate Prehension - food and test article taken into the mouth within two seconds.  2 - Delayed Prehension - food and test article taken into the mouth after two seconds |
| --- |
| 3 - No Prehension - test article and food mixture remaining after one minute |
| Y - Yes |
| Ya - Yes; consumed after one minute |

**Table S3: Acceptance in Phase II (Perod 2)- Individual Animal Data (continued)**


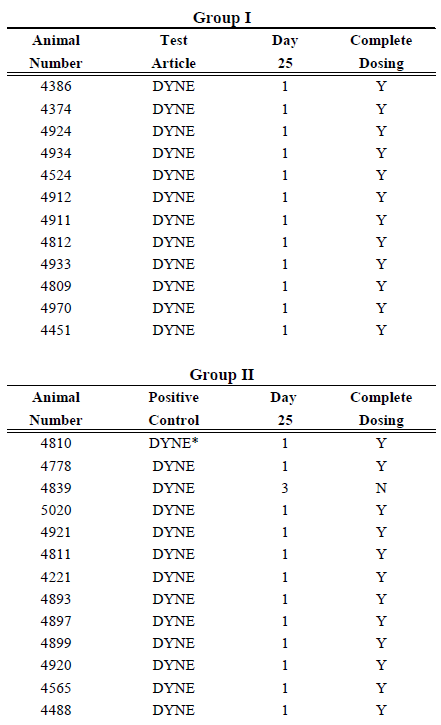


| * DYNE - Dyne High Calorie Liquid Diet Supplement |
| --- |
| 1 - Immediate Prehension - food and test article taken into the mouth within two seconds.  2 - Delayed Prehension - food and test article taken into the mouth after two seconds |
| 3 - No Prehension - test article and food mixture remaining after one minute |
| Y - Yes |
| Ya - Yes; consumed after one minute |

**Table S3: Acceptance in Phase II (Perod 2)- Individual Animal Data (continued)**


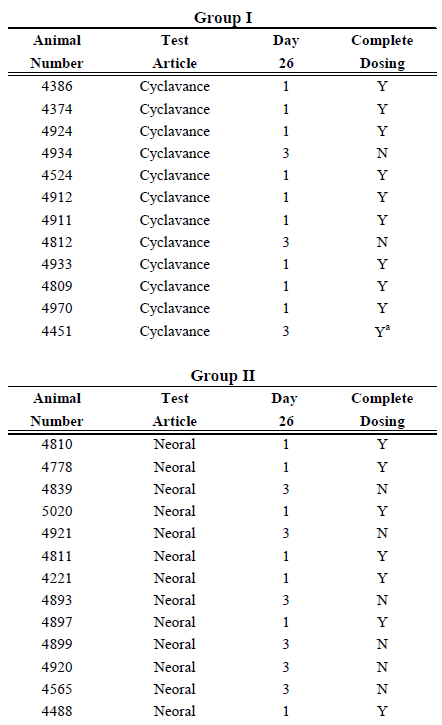


| 1 - Immediate Prehension - food and test article taken into the mouth within two seconds.  2 - Delayed Prehension - food and test article taken into the mouth after two seconds |
| --- |
| 3 - No Prehension - test article and food mixture remaining after one minute |
| Y - Yes |
| Ya - Yes; consumed after one minute |

**Table S3: Acceptance in Phase II (Perod 2)- Individual Animal Data (continued)**


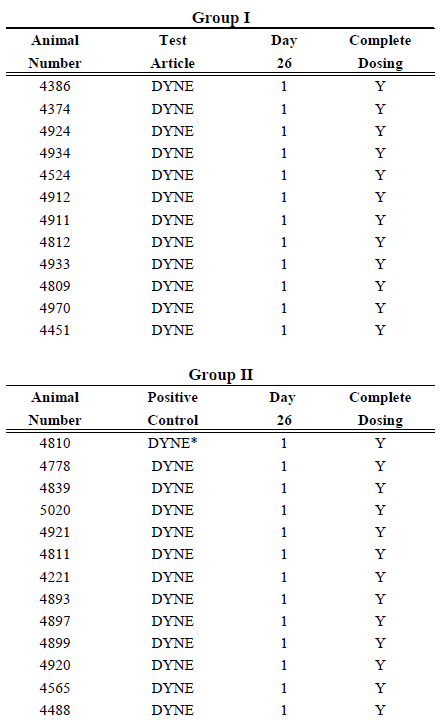


| * DYNE - Dyne High Calorie Liquid Diet Supplement |
| --- |
| 1 - Immediate Prehension - food and test article taken into the mouth within two seconds.  2 - Delayed Prehension - food and test article taken into the mouth after two seconds |
| 3 - No Prehension - test article and food mixture remaining after one minute |
| Y - Yes |
| Ya - Yes; consumed after one minute |

**Table S4: Individual Animal Body weights**


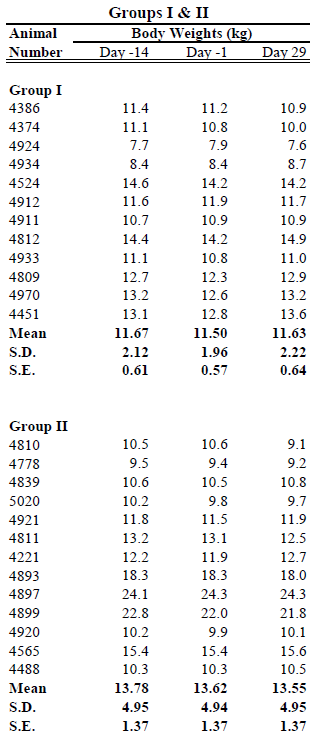


S.D. - Standard Deviation; S.E. - Standard Error

**Table S5: Physical Examination Data**


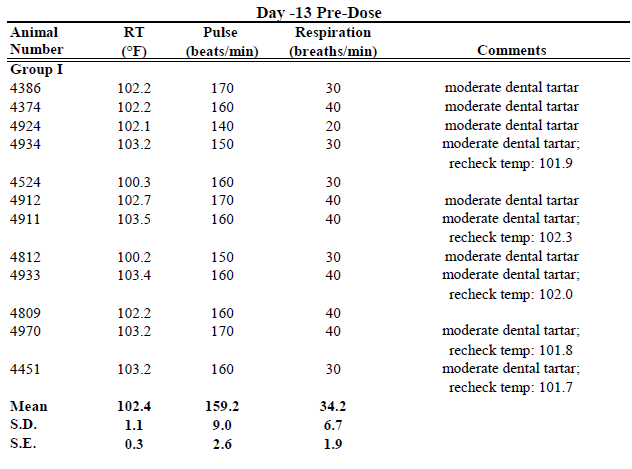


| Note: Higher temperatures for some animals were believed to be due to excitement. Temperatures were rechecked, as noted, when dogs were calmer. |
| --- |
| Note: The following items were examined for normal or abnormal: general appearance; body condition; behaviour; cardiovascular/pulmonary; ears, eyes and mouth; skin and hair; and study suitability. All parameters were marked normal unless otherwise noted. |
| RT - Rectal Temperature; min - minute; S.D. - Standard Deviation; S.E. - Standard Error |

**Table S5: Physical Examination Data (continued)**


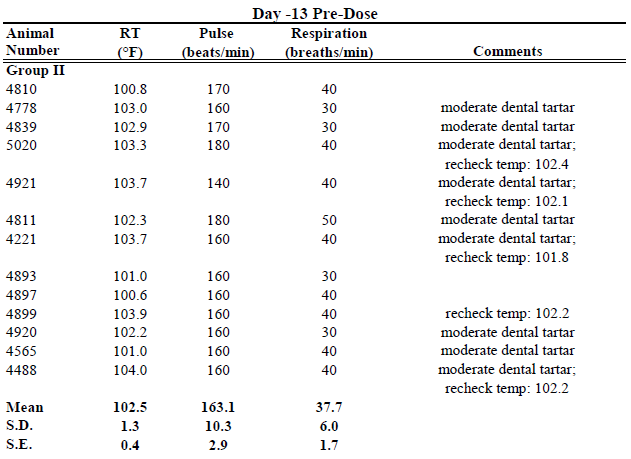


| Note: Higher temperatures for some animals were believed to be due to excitement. Temperatures were rechecked, as noted, when dogs were calmer. |
| --- |
| Note: The following items were examined for normal or abnormal: general appearance; body condition; behaviour; cardiovascular/pulmonary; ears, eyes and mouth; skin and hair; and study suitability. All parameters were marked normal unless otherwise noted. |
| RT - Rectal Temperature; min - minute; S.D. - Standard Deviation; S.E. - Standard Error |

**Table S5: Physical Examination Data (continued)**


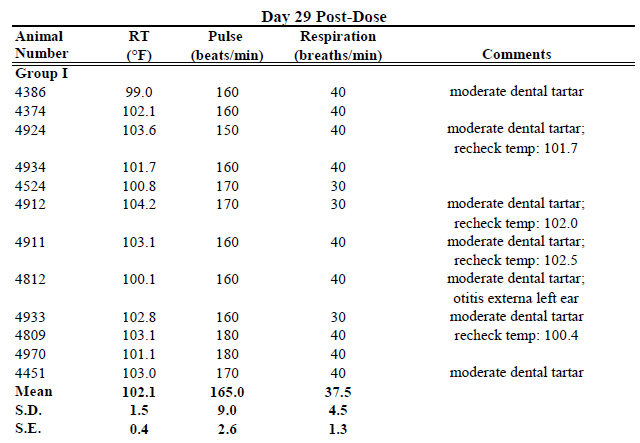


| Note: Higher temperatures for some animals were believed to be due to excitement. Temperatures were rechecked, as noted, when dogs were calmer. |
| --- |
| Note: The following items were examined for normal or abnormal: general appearance; body condition; behaviour; cardiovascular/pulmonary; ears, eyes and mouth; skin and hair; and study suitability. All parameters were marked normal unless otherwise noted. |
| RT - Rectal Temperature; min - minute; S.D. - Standard Deviation; S.E. - Standard Error |

**Table S5: Physical Examination Data (continued)**


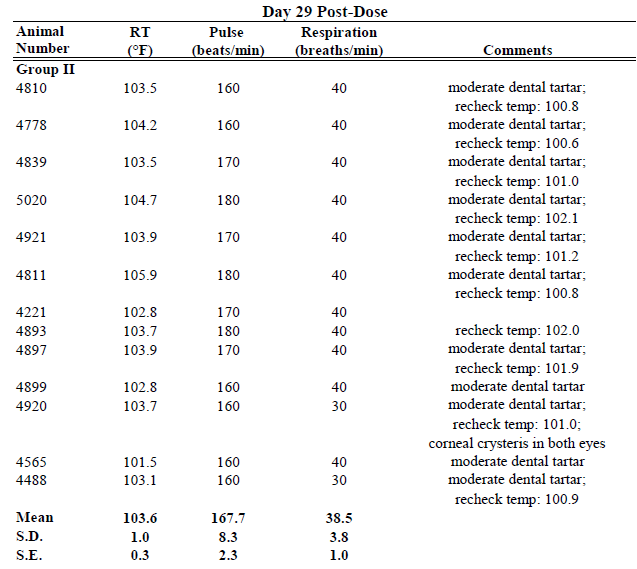


| Note: Higher temperatures for some animals were believed to be due to excitement. Temperatures were rechecked, as noted, when dogs were calmer. |
| --- |
| Note: The following items were examined for normal or abnormal: general appearance; body condition; behaviour; cardiovascular/pulmonary; ears, eyes and mouth; skin and hair; and study suitability. All parameters were marked normal unless otherwise noted. |
| RT - Rectal Temperature; min - minute; S.D. - Standard Deviation; S.E. - Standard Error |

**Table S6: Hematology Data**


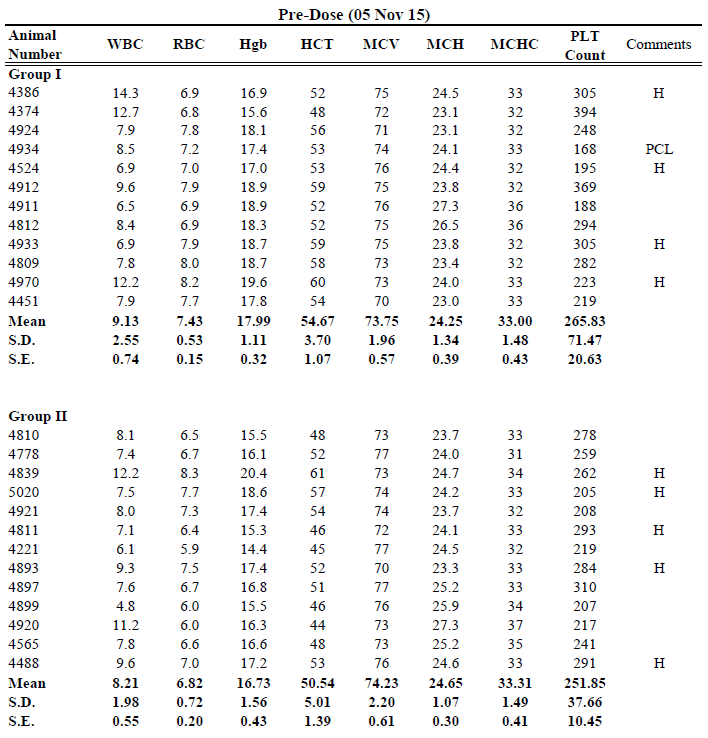


| H - Hemolysis 1+ - No significant interference |
| --- |
| PCL - Low platelet count reflects the minimum number due to platelet clumping. |
| S.D. - Standard Deviation, S.E. - Standard Error |

**Table S6: Hematology Data (continued)**


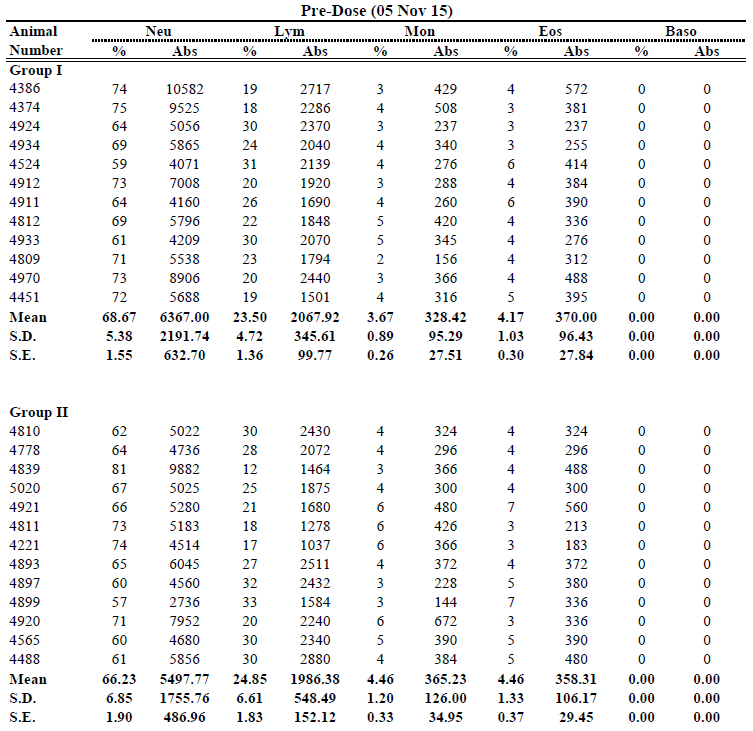


**Table S6: Hematology Data (continued)**


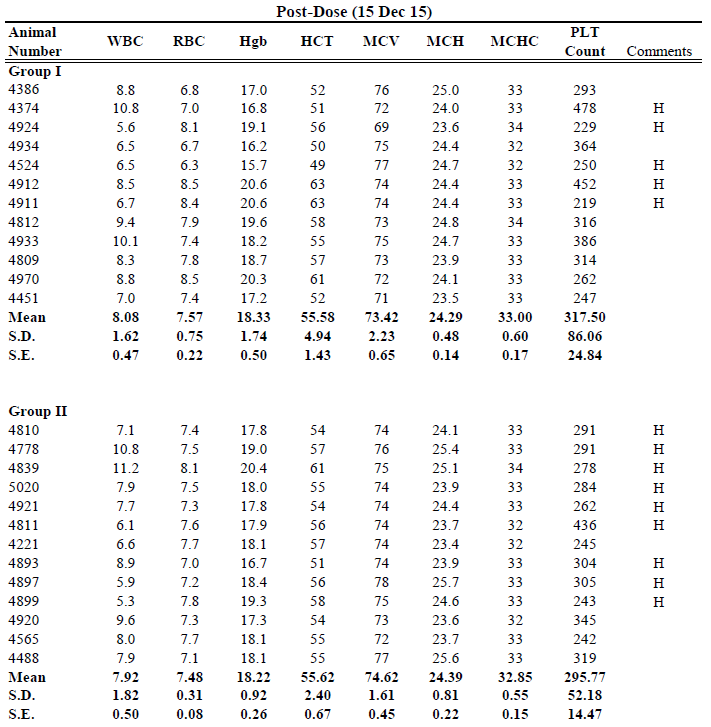


| H - Hemolysis 1+ - No significant interference |
| --- |
| PCL - Low platelet count reflects the minimum number due to platelet clumping. |
| S.D. - Standard Deviation, S.E. - Standard Error |

**Table S6: Hematology Data (continued)**


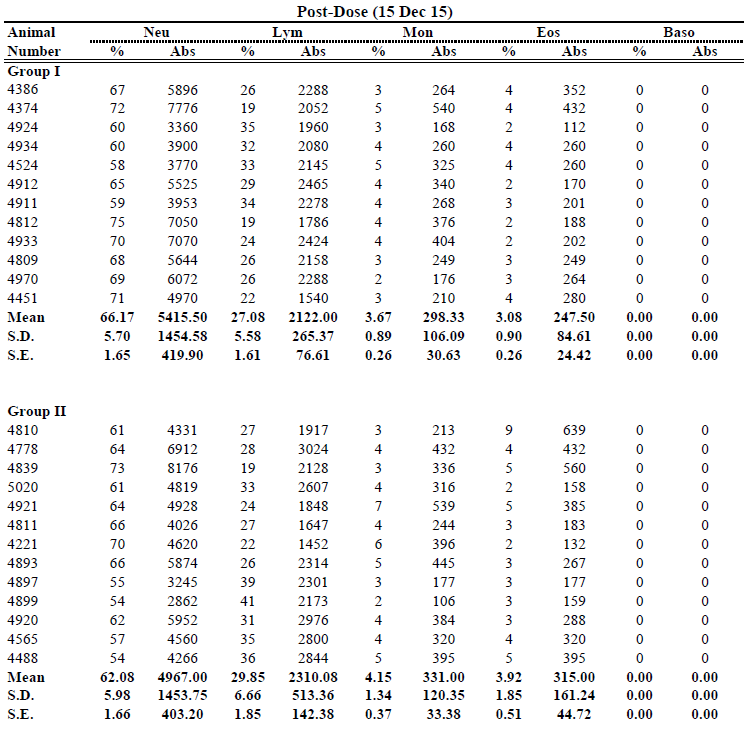


**Tabls S7: Serum Chemistry Data**


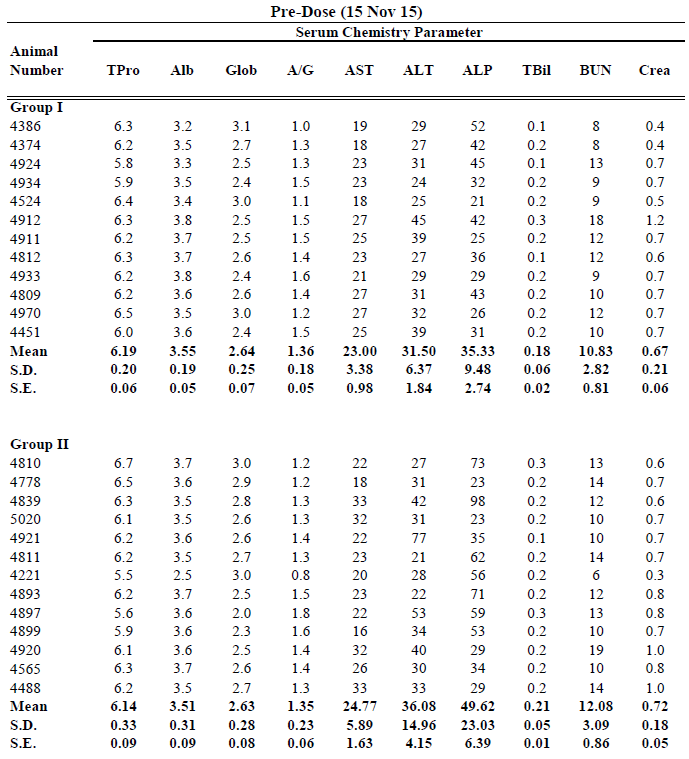


**Table S7: Serum Chemistry Data (continued)**


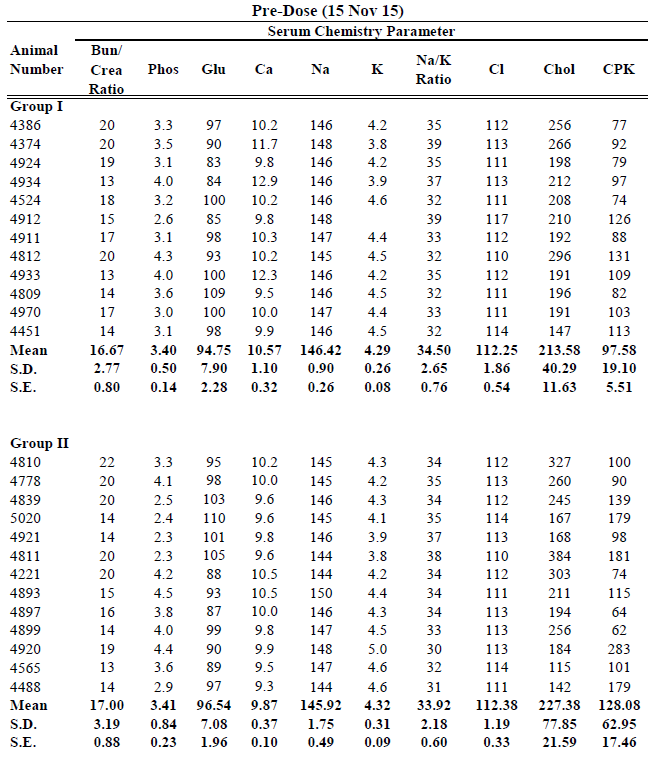


**Table S7: Serum Chemistry Data (continued)**


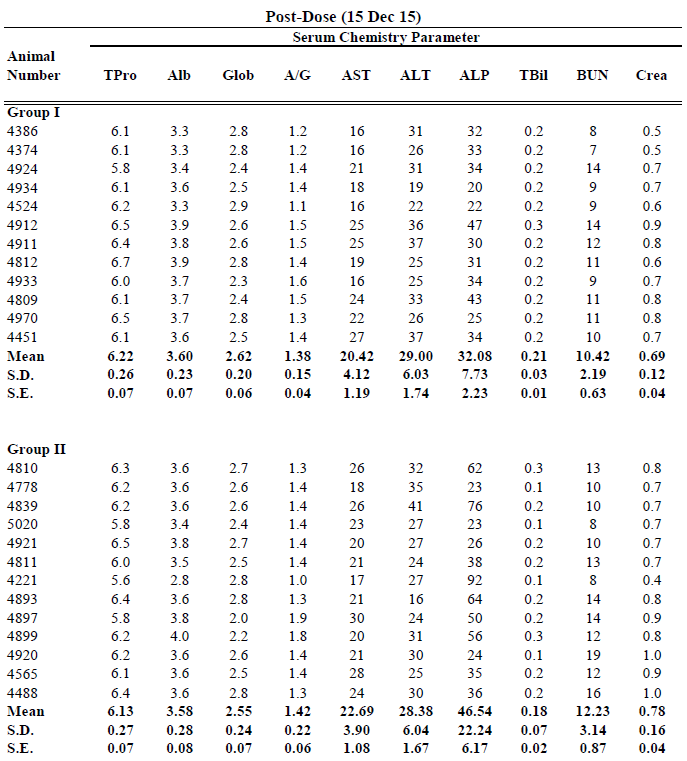


**Table S7: Serum Chemistry Data (continued)**


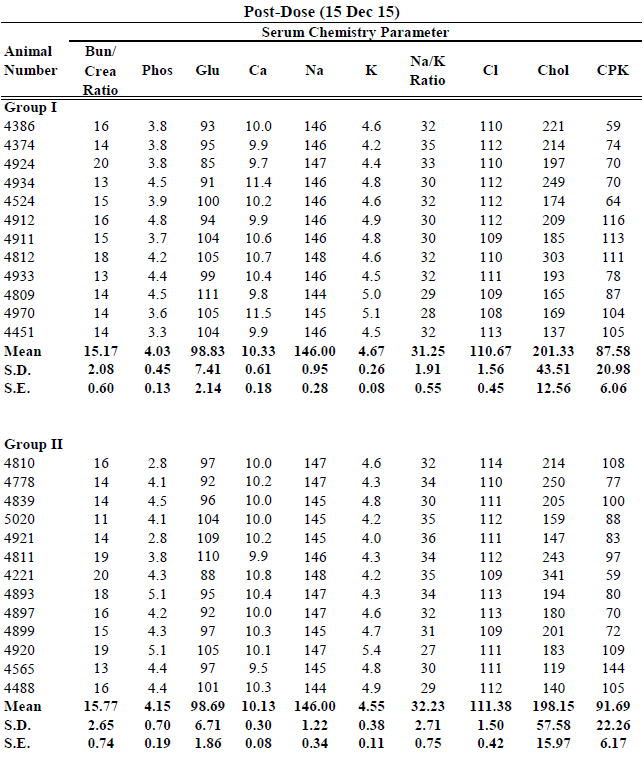


**Units of Hematology and Serum Chemistry Parameters**

| White Blood Cell Count (WBC) | 103/μL |
| --- | --- |
| Erythrocyte Count (RBC) | 106/μL |
| Hemoglobin (Hgb) | g/dL |
| Hematocrit (Hct) | % |
| Mean Corpuscular Volume (MCV) | fL |
| Mean Corpuscular Hemoglobin (MCH) | pg |
| Mean Corpuscular Hemoglobin Concentration (MCHC) | g/dL |
| Platelets (Plt) | 103/μL |
| Neutrophils (% Neut) | % |
| Lymphocytes (% Lymph) | % |
| Monocytes (% Mono) | % |
| Eosinophils (% Eosin) | % |
| Basophils (% Baso) | % |
| Total Protein (Tpro) | g/dL |
| Albumin (Alb) | g/dL |
| Globulin (Glob) | g/dL |
| Albumin/Globulin Ratio (A/G) | |
| Aspartate Aminotransferase (AST) | U/L |
| Alanine Aminotransferase (ALT) | U/L |
| Alkaline Phosphatase (ALP) | U/L |
| Total Bilirubin (TBil) | mg/dL |
| Urea Nitrogen (BUN) | mg/dL |
| Creatinine (Crea) | mg/dL |
| BUN/Creatinine Ratio (BUN/Crea) | |
| Phosphorus (Phos) | mg/dL |
| Glucose (Glu) | mg/dL |
| Calcium (Ca) | mg/dL |
| Sodium (Na) | mmol/L |
| Potassium (K) | mmol/L |
| Sodium Potassium Ratio (Na/K) | |
| Chloride (Cl) | mmol/L |
| Cholesterol (Chol) | mg/dL |
| Creatine phosphokinase (CPK) | U/L |
